# Food waste in residential aged care: A scoping review

**DOI:** 10.1111/1747-0080.70034

**Published:** 2025-08-29

**Authors:** Madeleine Roulston, Courtney Thompson, Fiona Pelly, Danielle Cave

**Affiliations:** ^1^ School of Health University of the Sunshine Coast Sippy Downs Queensland Australia

**Keywords:** elderly care, food wastage, long term care, nursing home, review

## Abstract

**Aims:**

The aim of this review was to explore the evidence available on food waste in residential aged care.

**Methods:**

This scoping review was conducted in accordance with the Joanna Briggs Institute methodology for scoping reviews. Peer‐reviewed literature was retrieved from six databases (PubMed, SCOPUS, CINAHL, Academic Search Premier, ProQuest Consumer Health, ProQuest Public Health), and grey literature was retrieved through Google up to January 2025. Results were screened by two independent reviewers. Data were extracted from relevant sources using a data extraction tool developed by the reviewers and were reported in tables and narrative text.

**Results:**

A total of 33 sources (20 peer‐reviewed and 13 grey literature sources) were included in this review. The majority reported an amount of waste, typically as a weight or percentage. Key findings of the review were that plate waste was the most measured food waste in residential aged care (*n* = 14) and that most studies used weighed methods (*n* = 10) or visual estimation (*n* = 6) to measure waste. Ten strategies to reduce food waste in this setting were identified, and only three studies conducted interventions to reduce food waste.

**Conclusion:**

This scoping review identified that plate waste was the most common type of food waste investigated and measured in this setting. However, measurement of other types of waste such as production and spoilage waste was limited and is an area for future research. This review provides several strategies to reduce food waste in this setting, and future research evaluating these strategies will be valuable for reducing total food waste in residential aged care.

## 1 INTRODUCTION

Globally, 1.3 billion tonnes of food go to waste each year, and this comes at an annual cost of almost US$940 billion to the global economy. Not only does this deplete valuable resources and money, but it also causes environmental harm and undermines sustainability efforts.^1^ Eliminating global food waste could potentially save 4.4 million tonnes of carbon dioxide (CO_2_) every year.^2^ The United Nations (UN) Sustainable Development Goal 12 is to ensure sustainable consumption and production patterns with the following objective; ‘by 2030, halve per capita global food waste at the retail and consumer levels and reduce food losses along production and supply chains’.^3^ Australia's National Food Waste Strategy was established in 2017, which sets a similar goal of halving Australia's food waste by 2030, and provides a framework to meet this target.^4^ In Australia alone, 7.6 million tonnes of food are wasted every year, costing the Australian economy A$36.6 billion per year.^5^ Reducing food waste is a matter of high priority globally, and requires collaboration from all of the sectors along the food supply and consumption chain.

Food waste refers to food that is intended for human consumption but is instead discarded, including spoilage waste (food that goes off and is not suitable for consumption), production waste (kitchen trimmings, and serving waste or leftovers that are never served), and plate waste (food that is served but not eaten).^6^ Food waste is generated across household, retail, hospitality, and healthcare sectors, and each sector faces their own unique challenges in waste reduction. The healthcare sector is known to make up 7% of Australia's total greenhouse gas emissions,^7^ and food waste in hospital and aged care settings contributes to this.^8^ In these settings, the impacts of food waste go beyond economic loss and environmental harm, as food waste can also be linked to the overall health of patients and residents.

Plate waste is often used as a means of determining a person's food and nutrient intake.^9^ Studies have demonstrated that high levels of plate waste are linked with poor dietary intake and lead to adverse outcomes such as malnutrition, slower recovery, and increased length of stay for hospital patients.^10^ Additionally, it has been shown that hospital patients who consume less than one quarter of the food provided are twice as likely to experience mortality during admission.^11^ Plate waste can also be an indication of satisfaction with the foodservice,^12^ and high levels of plate waste can indicate issues such as inappropriate portion size or a disconnect between the person's preferences and the food provided.^13^ These findings demonstrate the importance of monitoring plate waste in healthcare settings as it can be related to satisfaction with the food and subsequently food intake.

While research has been conducted to understand and reduce food waste in hospital settings, there is a paucity of literature on food waste in the residential aged care setting. Recent reviews of literature related to aged care foodservices address topics such as food fortification,^14^ perceptions and definitions of quality,^15^ and meal choice,^16^ but do not discuss food waste. A narrative review on plate waste in hospitals found a median plate waste of 30% across 32 studies.^9^ This review found that using a bulk food delivery system where meals are plated and served in the ward (e.g., buffet) had lower plate waste than those using a plated meal delivery system, as patients had choice in their meal at point of service and portion sizes could be individualized. However, residential aged care presents a unique setting with different challenges to hospitals, and it cannot be assumed that the same solutions would apply to this population group.

Food intake in the aged care population can be impacted by a range of individual factors due to aging, such as decreased appetite, taste changes, cognitive decline, and dysphagia.^17^ The strengthened Aged Care Quality Standards (strengthened Quality Standards) in Australia, which are scheduled to commence from 1st November 2025, have placed increased focus on food and nutrition. Standard 6 of the strengthened Quality Standards refers to ‘an enjoyable dining experience’ as an outcome and includes the expectation that food and drink served to aged care residents is nutritious, appetising and safe, and meets their needs and preferences. This involves making meal experiences pleasant, engaging and considering the preferences and cultural backgrounds of residents. The strengthened Quality Standards also acknowledge the impact that mealtimes can have on a person's quality of life.^18^ For aged care residents, mealtimes are not only necessary for nutrition, but also provide an opportunity for social interaction, and familiar foods can bring comfort and a sense of continuity.^19^, ^20^ High plate waste in residential aged care could indicate poor resident satisfaction, or that the meals are not meeting their individual needs.^21^ By identifying and addressing plate waste, aged care homes may have the potential to increase residents' quality of life through the food provision, demonstrate that they meet the requirements of the strengthened Quality Standards, and positively contribute to the UN's Sustainable Development Goals.

From a national and global perspective, the reduction of food waste is a key priority. Existing evidence in hospital settings emphasizes the implications of food waste on resource efficiency, financial burden, and environmental sustainability. As of 2023, there were just under 200 000 Australians living in residential aged care.^22^ By reducing food waste for this population, it would have positive impacts on the environment, the economy, and residents' health, satisfaction, and quality of life. Therefore, the objective of this scoping review was to explore and describe the existing body of knowledge on food waste in residential aged care, including the quantity of food waste, key sources of food waste, strategies to reduce food waste, and if particular foodservice systems result in less food waste. A scoping review was chosen for this study as this methodology can be used to explore the existing evidence on a topic, especially one that has not previously been explored in depth. The scoping review methodology assists with identifying gaps in the literature and future directions for research. The following research questions guided this review: What evidence is available on food waste in residential aged care?

The sub questions for this review were:

- How much food is wasted in residential aged care?
- What are the key sources of food waste in residential aged care?
- What foodservice systems result in less food waste?
- What are strategies to reduce food waste in this setting?

## 2 METHODS

This scoping review was conducted according to the Joanna Briggs Institute (JBI) methodology for scoping reviews and was reported using the Preferred Reporting Items for Systematic Reviews and Meta‐Analyses extension for Scoping Reviews (PRISMA‐ScR).^23^, ^24^ The scoping review protocol was registered with Open Science Framework (OSF) prior to the commencement of the review.^25^ The data extraction tool was developed by two reviewers and adapted from the JBI characteristics and results extraction instrument from the JBI Manual for Evidence Synthesis.^23^

In accordance with the JBI methodological guidance, the inclusion criteria for this review were guided by the Population, Concept, and Context mnemonic.^26^ The scoping review included both peer‐reviewed literature and grey literature, and only included sources relevant to the residential aged care setting. Sources examining the amount of food waste were included, as well as those that focused on strategies to prevent food waste. Sources that reported on food waste of any kind (spoilage, production, and plate waste), and had results of qualitative or quantitative nature were included. Studies conducted in hospitals were excluded. Studies considered for inclusion were primary research, qualitative, quantitative, observational, and interventional studies of any design or methodology published in peer‐reviewed journals. Grey literature sources, including government and research reports, theses and dissertations, action plans, and toolkits, were also included. Media sources such as blogs and newsletters were excluded. Sources from any country were included if they were available in English, and no date limits were applied. The databases and grey literature searches were conducted in August 2024, and the databases were searched again in January 2025 to include recent publications of relevance.

The search strategy was developed in consultation with a librarian. The final search strategy used in this review was: (‘food wast*’ OR ‘plate wast*’ OR ‘kitchen wast*’ OR ‘production wast*’) AND (‘aged care’ OR ‘elderly care’ OR ‘nursing home’ OR ‘long term care’ OR ‘long‐term care’ OR ‘aged care facilit*’ OR ‘senior housing’ OR ‘residential aged care facilit*’ OR ‘old age home’ OR ‘nursing facilit*’ OR ‘residential care’ OR ‘residential facilit*’ OR ‘care home’ OR ‘old peoples home’ OR ‘old people's home’). A total of six electronic databases were searched. They included PubMed, SCOPUS, CINAHL Ultimate, Academic Search Premier (EBSCO), and ProQuest (Consumer Health and Public Health databases). Following the database searches, all identified citations were imported into EndNote 21.4 and duplicates were removed. Then, the remaining results were uploaded into Covidence for screening. Titles and abstracts were screened by two independent reviewers for assessment against the inclusion criteria for the review, and the remaining relevant sources were retrieved in full. The review of all full text sources were performed in duplicate by the same two independent reviewers, and the sources were assessed against the inclusion criteria. Any conflicts that arose between the reviewers at each stage of the study selection process were resolved through discussion.

The grey literature searches were conducted through Google in the Google Chrome browser, utilizing private browsing through incognito mode. For these searches, each of the four food waste terms of the search strategy (‘food wast*’, ‘plate wast*’, ‘kitchen wast*’, and ‘production wast*’) was combined individually with each one of the aged care‐related terms (e.g., ‘aged care’, ‘nursing home’, etc.). For this grey literature search, truncation was replaced with a single letter (wast* became waste, and facilit* became facility). In total, this yielded 52 unique search combinations. The first 100 results for each search combination were screened for relevance by one independent reviewer. This involved reading the title and, if necessary, the summary of the link to determine if the source met the inclusion criteria for this review. The full texts of the remaining sources were reviewed in detail by the same reviewer and were chosen for inclusion or exclusion in the review. A second reviewer assisted with these final decisions.

Data from each of the 33 sources included in the scoping review were manually extracted by one reviewer using the data extraction tool. The data included details on the characteristics of the sources, study design, study aims, methods, and outcomes relevant to each of the review questions. Data on strategies to reduce food waste was collected from qualitative studies, as well as in the discussion sections of journal articles and in the grey literature. This data were recorded and organized using content analysis in accordance with the updated methodological guidance for conducting a JBI scoping review.^26^ The three phases of qualitative content analysis described by Elo and Kyngäs were used in this scoping review: (1) preparation; (2) organizing; and (3) reporting.^28^ Content analysis uses open coding of characteristics from the data into categories, and an inductive approach was used due to the paucity of evidence on the topic.^29^

## 3 RESULTS

**FIGURE 1:**
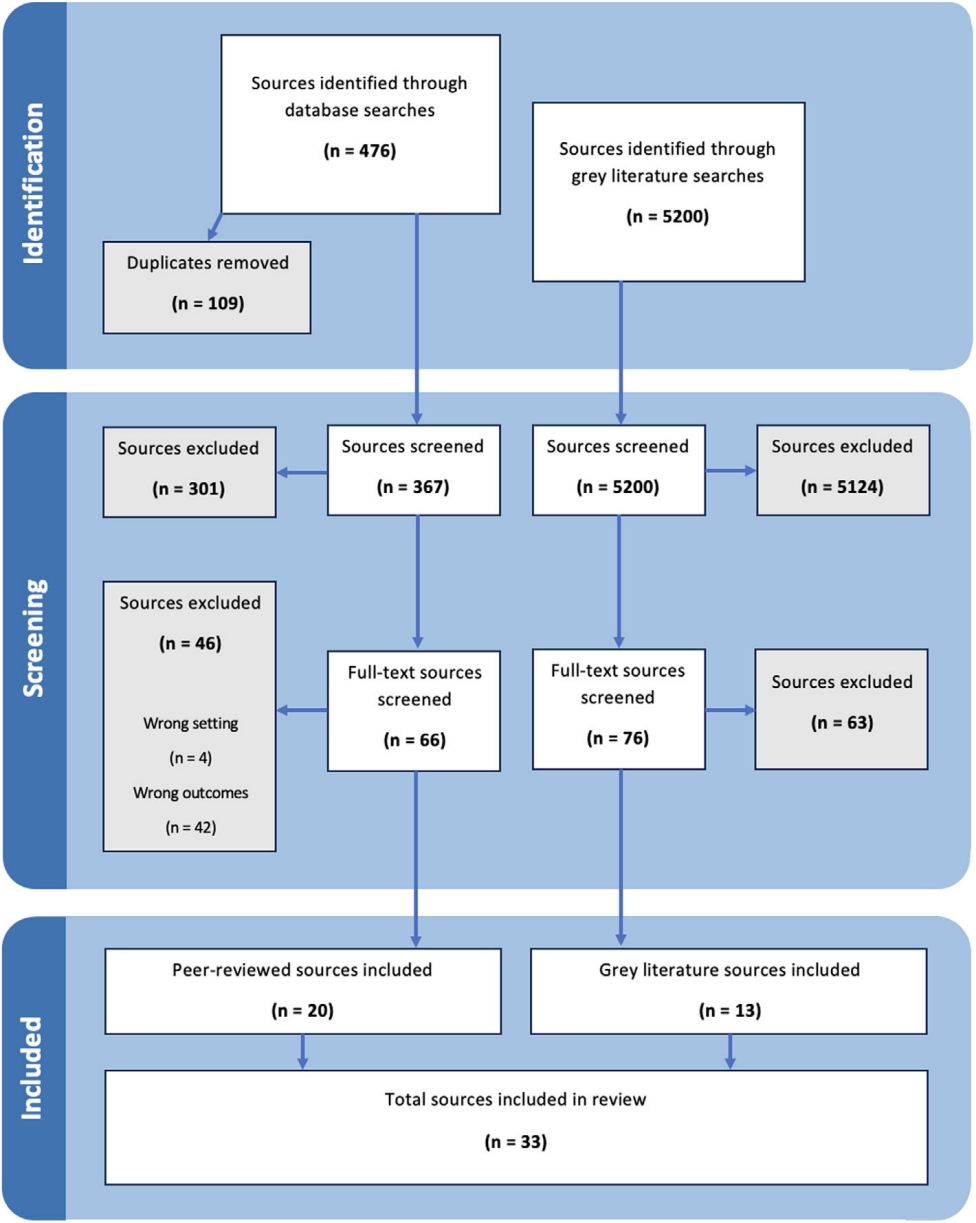
Flow chart of source selection for this scoping review adapted from PRISMA 2020 flow diagram for new systematic reviews.^27^

**FIGURE 2:**
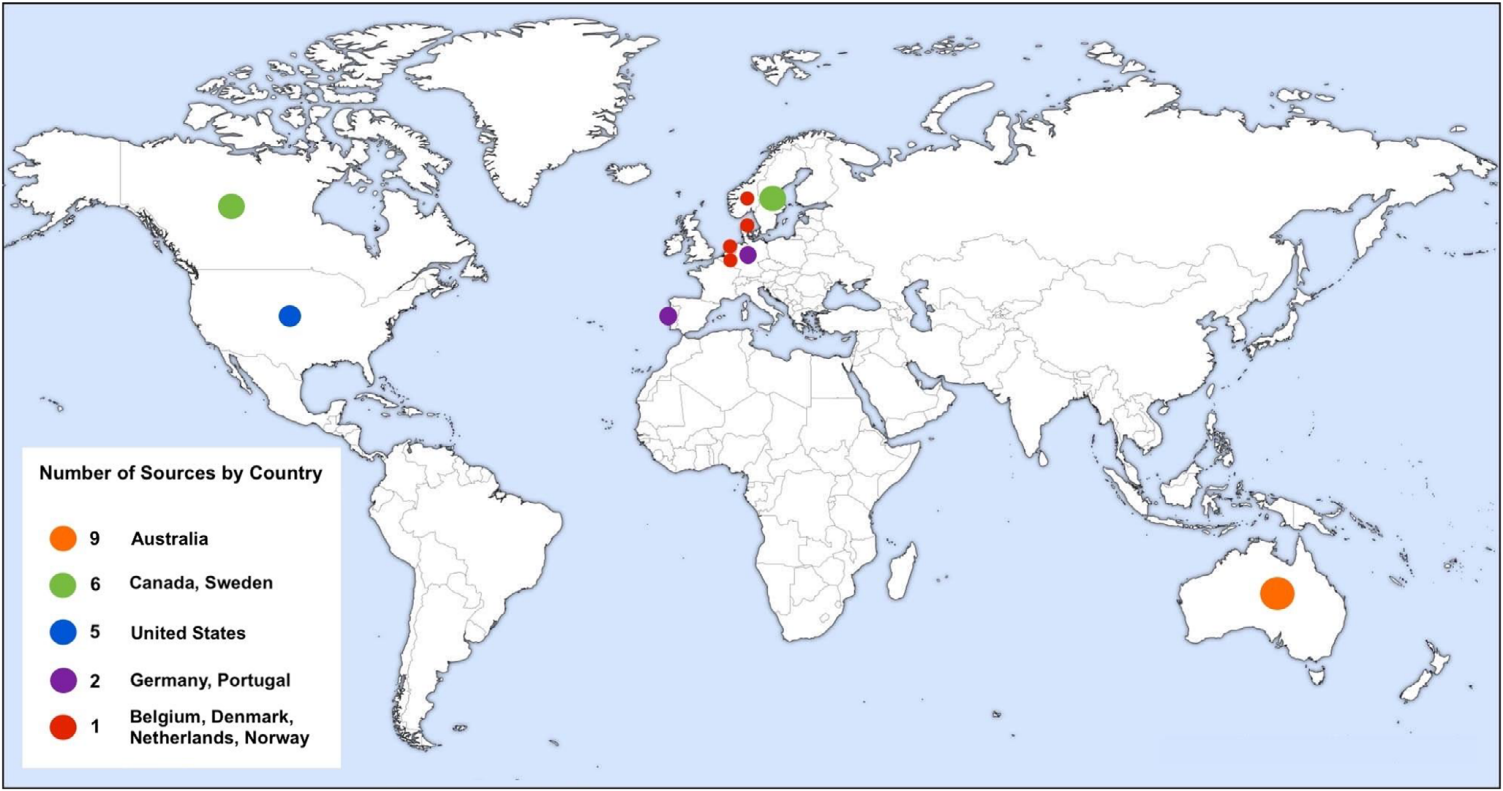
World heat map showing the number of sources included in this review by country.

A total of 33 sources (20 peer‐reviewed and 13 grey literature) were included in this review. The results of the search and screening process are presented in Figure 1. The sources originated from 10 countries, with over a quarter coming from Australia (*n* = 9) and the remaining from Europe and North America, as presented in Figure 2. Most peer‐reviewed studies had a cross‐sectional study design (*n* = 15). There were three quasi‐experimental studies,^30^, ^31^, ^32^ and two solely qualitative studies.^33^, ^34^ The most common types of grey literature sources included were reports (*n* = 3),^21^, ^35^, ^36^ and students' Masters research theses (*n* = 3).^37^, ^38^, ^39^ Other types of grey literature included were a better practice guide,^40^ pre‐print journal article,^41^ sustainability checklist,^42^ presentation slides,^43^ toolkit,^44^ conference abstract,^45^ and a book chapter.^46^ Across all sources, the most frequently reported type of waste was plate waste (*n* = 19), in comparison to production waste (*n* = 10), spoilage waste (*n* = 1), and total or unspecified food waste (*n* = 8). Food waste was the primary focus of 21 sources and secondary data (used to determine resident intake) in 12 sources. Source characteristics are summarized in Table 1.

**TABLE 1:** Characteristics of all sources reporting on food waste included in this review.

Author, year	Country	Setting, sample size	Source	Study design	Food waste as primary focus (*n* = 12) or secondary focus (*n* = 8) of study	Outcomes related to food waste	
						Amount (*n* = 18)	Strategies (*n* = 9)
Barth et al., 2019.^47^	Sweden	Aged care homes (*n* = 2)	Journal article	Cross‐sectional & qualitative	Primary	X	X
Eriksson et al., 2017.^48^	Sweden	Aged care homes (*n* = 4)	Journal article	Repeated cross‐sectional	Primary	X	
Eriksson et al., 2023.^49^	Sweden	Aged care homes (*n* = 5)	Journal article	Repeated cross‐sectional	Primary	X	X
Gales and Roy‐Poirier, 2009.^50^	Canada	Aged care homes (*n* = 2) Residents (*n* = 100; *n* = 57)	Journal article	Cross‐sectional	Secondary	X	
Giampaoli and Khanna, 2000.^51^	USA	Aged care home (*n* = 1) Residents (*n* = 56–59)	Journal article	Cross‐sectional	Secondary	X	
Grieger and Nowson, 2005.^52^	Australia	Aged care home (*n* = 1) Residents (*n* = 66)	Journal article	Cross‐sectional	Secondary	X	
Grieger and Nowson, 2007.^53^	Australia	Aged care home (*n* = 1)	Journal article	Cross‐sectional	Secondary	X	
Hansen and Derdowski, 2020.^30^	Norway	Residents (*n* = 12)	Journal article	Quasi‐experimental	Primary	X	
Hoefnagels et al., 2023.^33^	Netherlands	Aged care homes (*n* = 2)	Journal article	Qualitative	Secondary		X
Huang and Shanklin, 2008.^54^	USA	Aged care homes (*n* = 5)	Journal article	Cross‐sectional	Secondary	X	X
Iuliano et al., 2013.^55^	Australia	Aged care homes (*n* = 18)	Journal article	Cross‐sectional	Secondary	X	
Iuliano et al., 2017.^56^	Australia	Aged care homes (*n* = 21)	Journal article	Cross‐sectional	Secondary	X	
Liu and Oulton, 2016.^57^	Canada	Residents (*n* = 52)	Journal article	Cross‐sectional	Primary	X	
Martins et al., 2021.^58^	Portugal	Aged care home (*n* = 1)	Journal article	Cross‐sectional	Primary	X	X
Malefors et al., 2019.^59^	Sweden & Germany	Aged care homes in Sweden (*n* = 20) Aged care homes in Germany (*n* = 42)	Journal article	Repeated cross‐sectional	Primary	X	
McAdams et al., 2023.^60^	Canada	Aged care homes (*n* = 2)	Journal article	Cross‐sectional & qualitative	Primary	X	X
Miller et al., 2024.^61^	Canada	Residents (*n* = 33)	Journal article	Cross‐sectional	Primary	X	X
Ofei et al., 2015.^34^	Denmark	Aged care homes (*n* = 2)	Journal article	Qualitative	Primary		X
Strotmann et al., 2017.^31^	Germany	Aged care home (*n* = 1) Residents (*n* = 74)	Journal article	Quasi‐experimental	Primary	X	X
Wheeler et al., 2025.^32^	Australia	Aged care home (*n* = 1) Residents (*n* = 36)	Journal article	Quasi‐experimental	Primary	X	
Aged and Community Services Australia (ACSA), 2018.^40^	Australia	Aged care home (*n* = 1)	Grey literature	Better practice guide	Secondary	X	X
Australian Government, 2023.^21^	Australia	Residents (*n* = 365)	Grey literature	Report	Secondary	X	X
Bernaert et al., 2024.^41^	Belgium	Residents (*n* = 247)	Grey literature	Pre‐print journal article	Primary	X	X
Byles et al., 2009.^35^	Australia	Aged care homes (*n* = 5)	Grey literature	Report	Secondary	X	
Connecticut Department of Energy and Environmental Protection, 2020.^42^	USA	Not applicable	Grey literature	Sustainability checklist	Primary		X
Fritz, 2019.^43^	Sweden	Not reported	Grey literature	Presentation slides (based on a report published in Swedish)	Primary	X	
Golder, 1984.^37^	USA	Aged care home (*n* = 1)	Grey literature	Master's thesis	Primary		X
Imteaz, 2022.^38^	Sweden	Aged care homes (*n* = 20)	Grey literature	Master's thesis	Primary	X	
Love Food Hate Waste NSW, 2020.^44^	Australia	Not reported	Grey literature	Toolkit	Primary	X	X
McAdams, 2021.^45^	Canada	Aged care homes (*n* = 2)	Grey literature	Conference abstract	Primary	X	
Office of the Auditor General of Ontario, 2019.^36^	Canada	Aged care homes (*n* = 59)	Grey literature	Report	Secondary	X	X
Pereira et al., 2025.^46^	Portugal	Residents (*n* = 13)	Grey literature	Book chapter	Primary	X	X
Robbins, 2019.^39^	USA	Aged care home (*n* = 1) Residents (*n* = 100)	Grey literature	Master's thesis	Primary	X	X

The amount of food waste was the most common outcome, reported by 29 of the 33 included sources, which is presented in Table 2. In the peer‐reviewed sources, food waste was measured using validated tools including weighing (*n* = 10),^62^ visual estimation (*n* = 6),^63^ and photography (*n* = 1).^64^ In the case of total food waste being reported, this was typically weighed, and when production waste was quantified, it was invariably weighed. The sources in this review were largely heterogeneous in the way that they reported waste, including a percentage of waste from food intended for human consumption, a weight (grams or kilograms), or a monetary value. Within these categories, there was great variation in the units used. For example, as shown in the ‘percentage’ column in Table 2, some sources report a percentage of plate waste, while others report the percentage of total food waste that is made up of plate waste. In the ‘weight’ column, some amounts were reported in g/resident/day, while other sources reported g/portion or kg/day.

**TABLE 2:** Quantifiable outcomes for food waste from included sources (*n* = 28).

Study/source	Type of food waste	Waste amount (%)	Waste amount (weight)	Cost of waste	Additional information	Method of waste measurement
Barth et al., 2019.^47^	Total/unspecified food waste		0–5 kg/day		Reported by ~20% of respondents.	Not reported.
			5–10 kg/day		Reported by >50% of respondents.	
			15–20 kg/day		Reported by ~20% of respondents.	
Eriksson et al., 2017.^48^	Plate waste	23% (range of 13%–34%) 33% of total food waste	75 g/portion (range of 33–131 g)			Weighed.^a^
	Serving waste	64% of total food waste				
	‘Other’ food waste	3% of total food waste				
Eriksson et al., 2023.^49^	Total/unspecified food waste		32–48 g/guest/day			Weighed.^a^
Gales and Roy‐Poirier, 2009.^50^	Total/unspecified food waste	11% of all waste in facility Y 1% of all waste in facility X				Exact methods for food waste measurement unclear—mass balance of facilities conducted.
Giampaoli and Khanna, 2000.^51^	Plate waste	56% of vegetables 59% of starch 30% of entrees 36% of dessert			Data collected over seven lunch meals. Census for the data collection period ranged from 56 to 59 residents.	Visual estimation.^b^
Grieger and Nowson, 2005.^52^	Plate waste	23% at main meals 17% at mid‐meals			Mean plate waste from a single, whole day of data collection.	Visual plate waste rating scale.^b^
Grieger and Nowson, 2007.^53^	Plate waste	17% of total energy 16% of energy at main meals 22% of energy at mid‐meals			Mean energy wasted via plate waste from three single, whole day assessments.	Visual plate waste rating scale.^b^
Hansen and Derdowski, 2020.^30^	Plate waste	26% on white plate 10% on yellow well, red lip, red ring 22% on white well, green lip, blue rim 9% on white well, yellow rim and red ring				Visual estimation using side by side photographs of plates before and after meals.^c^
Huang and Shanklin, 2008.^54^	Plate waste	15%			Note that this waste percentage is taken from the given result ‘an average of 85% of the food served was consumed by residents in the five facilities’.	Weighed.^a^
Iuliano et al., 2013.^55^	Plate waste	0%–15%			Data collected using. 3–6 day weighed food records. Study reports daily mean number of serves of each food groups provided, wasted and consumed for men, women and all.^d^	Weighed.^a^
Iuliano et al., 2017.^56^	Plate waste				Data collected on two random days. Study reports daily mean number of serves of each food groups provided, wasted and consumed for male and female residents.^d^	Validated visual estimation.^b^
Liu and Oulton, 2016.^57^	Plate waste/point of service waste		37.1 kg/day (solid food waste) 16.6 kg/day (fluid waste)	CA$57000 per year (AU$62962.20) 40% of annual food budget (estimated)	Fluid waste did not include unserved fluid which did not have to be discarded. Data collected over a two‐week period.	Weighed.^a^
Martins et al., 2021.^58^	Plate waste	12% of prepared food	50 g/resident/meal 166 kg total during study period	€29 262 per year (AU$50608.92)	Data collected over 15 consecutive days, at lunch and dinner. Study gives data for mean % + SD, and minimum‐maximum range for the each meal component.^d^	Weighed.^a^
	Leftovers	24.1% of prepared food	150 g/resident/meal	€77 849 per year (AU$134640.62)		
	Total food waste	36.1% of meals was wasted		€107 112 per year (AU$185251.28)		
Malefors et al., 2019.^59^	Total food waste	20.6%	129 g/portion		Data obtained over 2155 quantification days.	Weighed.^a^
McAdams et al., 2023.^60^	Total food waste	58%	209.75 kg over study period 570 g/resident/meal	CA$5.43 per resident (AU$6)	Data collected over 14 meal periods (lunches and dinners only).	Weighed.^a^
	Leftover waste	76% of total food waste				
	Plate waste	24% of total food waste.				
Miller et al., 2024.^61^	Plate waste	28% on average 25% at breakfast 31% at lunch 27% at dinner 35% at afternoon snack 45% at evening snack			Comprehensive 3‐day waste audit. Study also reports food waste for texture modified foods (not shown in this table).	Visual estimation.^b^
Strotmann et al., 2017.^31^	Total food waste	21.4% pre‐intervention 13.4% post‐intervention			2 week data collection period before implementing measures to reduce food waste, then another data collection. Study gives plate waste and serving loss data pre and post intervention for each meal.^d^	Weighed.^a^
Wheeler et al., 2025.^32^	Plate waste	Total: 21% pre‐intervention 15% post‐intervention			Data were collected across lunch and dinner meals, Monday–Friday for three and a half weeks pre‐intervention, and six weeks during intervention.	Visual estimation (plate waste).^b^
	Production waste		Total: 55 g pre‐intervention 90 g post‐intervention		Grams per resident per day. Study gives pre and post intervention data for each meal component.^d^	Weighed (production waste).^a^
Aged and Community Services Australia (ACSA), 2018.^40^	Total food waste	19% of all waste	7.3 tonnes/year (2011) 5.1 tonnes/year (2017)		Tonnes/year data from a waste and recycling review.	
Bernaert et al., 2024.^41^	Plate waste	10.9%	112.3 g/resident/day		Monitored over 5 days at breakfast, lunch and dinner.	Plate waste at breakfast and dinner were measured with visual estimation. Plate waste and production waste at lunch were weighed.
	Preparation waste	37.8%	192.6 g/resident/day			
Byles et al., 2009.^35^	Plate waste	7.5%–24% at first measurement point 4%–15% at second measurement point			Data measured at two points in time. Mean waste in percentage. Study gives data for mean waste at each meal.^d^	Unclear whether visual or weighed, ‘nutrition assessors recorded the amount of food left on plates after meals’
Fritz, 2019.^43^	Kitchen waste		11 g/person (median) Ranged from 0.2–99 g		Data collected via voluntary survey sent to meal managers in Sweden's municipalities (self‐reported data from lunches over 5 days).	
	Serving waste		85 g/person (median) Ranged from 19 to 214 g			
	Plate waste		29 g/person (median) Ranged from 7 to 156 g			
Imteaz, 2022.^38^	Total food waste		121.5 g/portion on average from 2013 to 2020. Median waste per portion, by year: 77 g in 2013 107 g in 2014 85 g in 2015 107 g in 2016 101 g in 2017 98 g in 2018 125 g in 2020		Data were recorded by municipalities themselves and was compiled for the study.	
Love Food Hate Waste NSW, 2020.^44^	Total food waste	11.2% of the aged care sector's total waste	3.5 kg/resident/week	AU$1000 per facility, per week	Data from NSW, Australia.	
McAdams, 2021.^45^	Total food waste	>50%			Data including food waste across all aspects of the food delivery system.	
Office of the Auditor General of Ontario, 2019.^36^	Plate waste	25%–60%		CA$2.48 per resident, per day (AU$2.74)	Data are an estimation by management and food service workers.	
Pereira et al., 2025.^46^	Plate waste Leftovers	Total: 11.8 ± 2.9% Total: 29.7 ± 8.8%	Total: 0.7 ± 0.2 kg Total 2.6 ± 1.0 kg		Data from lunch meals over 20 days. Kg total per lunch period (mean ± SD). Study gives data for each meal component.^d^	Weighed
Robbins, 2019.^39^	Total food waste	25%	63.75 pounds total during study period 31 pounds of starch foods 20 pounds of vegetables 6.25 pounds of entrees	20% of all funds spent on food were wasted US$85.85 total (AU$136.80) US$43.66 for starches (AU$69.57) US$19.67 for vegetables (AU$31.34) US$22.52 for entrees (AU$35.88)	Data from 5 lunch periods.	Weighed

*Note*: Monetary values reported in currencies other than Australian dollars include a conversion (AU$) that is correct as of March 2025.

^a^ Validated weighed method for measuring plate waste.^62^

^b^ Validated visual estimation method for measuring plate waste.^63^

^c^ Validated method using photography for measuring plate waste.^64^

^d^ Sources which have detailed information on food waste amount by gender, mealtime, meal component, or food group are not shown in this table.

Seven sources included in the review reported food waste by food group or meal component. Within these sources, the food groups and meal components reported on varied. Two sources reported waste for each of the five food groups,^55^, ^56^ and five reported by meal component such as main dish, soup, vegetables, starch, etc.^32^, ^35^, ^39^, ^46^, ^51^ There wasn't enough detail provided in the included sources to gather information on key types or sources of food waste; where present, this information was usually given as a percentage and is shown in Table 2. Sources rarely provided detail on the foodservice system present in their study, resulting in one of the research questions going unanswered: whether particular foodservice systems result in less food waste. The collection of data in Table 2 provides a broad overview of food waste data in residential aged care, across various types of waste and methods of measurement.

Out of the three studies included in this review that conducted interventions, two did so with the primary goal of reducing food waste pre and post implementation.^30^, ^31^ Both demonstrated successful food waste reduction strategies. One study employed a participatory approach and involved employees in the process of developing and implementing waste reduction measures. This led to the development of a range of measures to improve resource efficiency, covering five key topics: information, communication, products, processes, and customer needs. Overall, these measures resulted in a reduction of food waste from 21.4% to 13.4%.^31^ Another study tested one specific strategy to reduce plate waste, which was to redesign dinnerware. They tested white porcelain plates (most used in residential aged care) against three different colors and designs of plates. The white plate had an average plate waste measure of 26%, while the plate design with the lowest average—a white well, yellow rim, and red ring around the edge—had just 9%.^30^ The third study was focused on increasing meal choice for residents and implemented changes to the menu and meal ordering system that allowed residents to place their orders at point of service during the mealtime and choose from one chef special, salad, sandwiches (fresh or toasted) and a list of six hot options, utilizing a cook chill system with readymade meals. This system did result in improved resident satisfaction and reduced plate waste; however, it also showed an increase in production waste.^32^

Ten strategies to address and reduce food waste in the residential aged care setting were identified through inductive content analysis and are shown in Table 3. They are organised into four categories: monitor food waste, increase awareness of food waste, divert food waste from landfill, and improve menu planning. Sources noted the importance of raising awareness of food waste among residents and their families, as well as staff.^58^ It was also emphasised that all staff members had a role to play in the reduction of food waste.^42^ Disconnection from resident preferences was often attributed to policy that set out a predetermined portion size or nutrient intake. This was found to contribute to waste as portions were often too large and were not flexible in response to residents' fluctuating appetites. Poor forecasting was also a major contributor to food waste, with one source reporting that ordering buffers were the largest contributor to food waste. This refers to foodservice staff preparing more meals than the number of residents to provide choice, which can result in significant production waste. The study gives an example of poor forecasting in which 30 entrees were produced for 22 people, resulting in a large amount of food waste before any food was served.^60^

**TABLE 3:** Strategies to reduce food waste in residential aged care identified in sources included in this review.

Categories	Strategies	Sources
Monitor food waste	Set goals	Connecticut Department of Energy and Environmental Protection, 2020.^42^
		Love Food Hate Waste NSW, 2020.^44^
		Eriksson et al., 2023.^49^
	Benchmark	Connecticut Department of Energy and Environmental Protection, 2020.^42^
		Love Food Hate Waste NSW, 2020.^44^
		Martins et al., 2021.^58^
		Ofei et al., 2015.^34^
		Strottman et al., 2017.^31^
Increase awareness of food waste	Improve communication	Connecticut Department of Energy and Environmental Protection, 2020.^42^
		Love Food Hate Waste NSW, 2020.^44^ Barth et al., 2019.^47^
		Eriksson et al., 2023.^49^
		Liz Martins et al., 2021.^58^
		Ofei et al., 2015.^34^
		Strottman et al., 2017.^31^
Divert food waste from landfill	Donate food	Aged and Community Services Australia (ACSA), 2018.^40^
		Connecticut Department of Energy and Environmental Protection, 2020.^42^
		Office of the Auditor General of Ontario, 2019.^36^
		Ofei et al., 2015.^34^
	Compost	Aged and Community Services Australia (ACSA), 2018.^40^
		Connecticut Department of Energy and Environmental Protection, 2020.^42^
		Office of the Auditor General of Ontario, 2019.^36^
	Repurpose leftovers	Connecticut Department of Energy and Environmental Protection, 2020.^42^
		Eriksson et al., 2023.^49^
		Ofei et al., 2015.^34^
Improve menu planning	Reduce portion sizes	Bernaert et al., 2024.^41^
		Office of the Auditor General of Ontario, 2019.^36^
		Eriksson et al., 2023.^49^
		Miller et al., 2024.^61^
		Ofei et al., 2015.^34^
		Strottman et al., 2017.^31^
	Align policy with resident preferences	Connecticut Department of Energy and Environmental Protection, 2020.^42^
		Goldner, 1984.^37^
		Robbins, 2019.^39^
		Martins et al., 2021.^58^
		McAdams et al., 2023.^60^
		Ofei et al., 2015.^34^
		Strottman et al., 2017.^31^
		Pereira et al., 2025.^46^
	Flexible forecasting systems	Barth et al., 2019.^47^
		Hoefnagels et al., 2023.^33^
		Ofei et al., 2015.^34^
		Strottman et al., 2017.^31^
	Appropriate food texture	Australian Government, 2023.^21^
		Goldner, 1984.^37^
		Huang and Shanklin, 2008.^54^
		Strottman et al., 2017.^31^

## 4 DISCUSSION

This scoping review explored the existing body of knowledge on food waste in residential aged care, as well as identified strategies to help reduce food waste in this setting. The type of food waste most frequently reported was plate waste, similar to the existing literature in hospital settings.^9^ This could be due to the relationship between plate waste, patient health outcomes, and patient satisfaction, which makes it an important output of the foodservice system across all healthcare settings. It may also be due to plate waste being easier to quantify, with the existence of validated data collection methods such as visual estimation, photography, and weighing using electronic scales.^62^, ^63^, ^64^ However, in many cases, plate waste data were reported in a source where the primary focus was not on food waste itself, but it was instead measured to determine residents' food intake. Food intake and waste are important measures in residential aged care, where food and meal quality are the most discussed factors for foodservice quality.^15^ Still, there is further opportunity to explore plate waste itself in this setting and its relationship with resident wellbeing and outcomes.

There was a distinct lack of studies that tested interventions to reduce food waste in residential aged care. Only three of the included studies applied an intervention, and two were primarily focused on waste reduction, both of which demonstrated the success of targeted strategies to reduce waste.^30^, ^31^ These two sources emphasized the potential for practical, cost‐effective waste reduction strategies in this setting. The study by Hansen et al. that focused on redesigning dinnerware presents a somewhat simple change that reduced plate waste from 26% to as little as 9%.^30^ Previous studies have found that high‐contrast dinnerware (e.g., blue) may increase food intake for aged care residents living with dementia when compared with standard white dinnerware.^65^, ^66^, ^67^ This would likely be a feasible change in most residential aged care homes, and based on those findings, could have significant impacts. In another study, the participatory approach used by Strottman et al. was found to be successful in the reduction of plate and production waste. In this study, staff were involved in the development and implementation of the measures that led to an 8% reduction in total waste in the aged care home.^31^ This is in line with one of the suggested strategies to reduce waste across all sources included in the review, which was to increase awareness of food waste. This includes increasing communication and engagement with staff at all levels, residents, and their families about the food waste issue. The sentiment that it takes a combined effort to reduce waste and that everyone has a role to play in food waste reduction was considered essential. The causes of food waste, and therefore the strategies to address it, are often unique to each aged care home, so local quality assessment processes to identify sources of waste and appropriate solutions are the most effective.

The study by Wheeler et al. focused on increasing meal choice for people living in residential aged care.^32^ Pre and post‐implementation, data on the outcome measures of food waste, resident satisfaction, and foodservice costs were collected. The increased choice resulted in improved resident satisfaction and decreased the average total plate waste from 21% to 15%. This is a positive change that demonstrates a relationship between resident satisfaction and plate waste. However, the changes to the foodservice system also resulted in an increase in production waste and foodservice costs. Thus, the study concluded that further work is needed to investigate how production waste and costs can be addressed when offering increased choice. These findings highlight the importance of measuring all aspects of a foodservice system when making changes to the foodservice model, as there may be unintended consequences such as increases in food waste elsewhere in the system.

This review also identified several other strategies that offer promising directions for residential aged care homes looking to reduce food waste. The importance of monitoring food waste was a key point raised in six sources, including government resources aimed at assisting aged care homes to reduce waste.^42^, ^44^ Establishing a food waste baseline is suggested as an important first step in the reduction of waste.^68^ Diverting food waste from landfill was also identified, which is in alignment with the Food Recovery Hierarchy where landfill/incineration is the least preferred option in comparison to diverting waste or reducing it at the source.^69^ Lastly, improving menu planning by reducing portion sizes, aligning policy with resident preferences, using flexible forecasting systems, and ensuring appropriate food texture were highlighted as opportunities to decrease waste and improve patient satisfaction. The success of these strategies has not been evaluated in the literature, but they may help reduce food waste if they are tailored to the aged care home they are implemented in.

Residential aged care settings are complex and require careful consideration when implementing waste reduction strategies. The sources in this review identified common challenges such as rigid policies that mandate portion size. While these policies are put in place to ensure residents' needs are met, they lack flexibility for individual resident‐specific needs and preferences. In contrast to hospital settings, where patients are generally short‐stay and acutely ill, residential aged care is a setting where there is an opportunity to explore residents' food preferences and better cater to individual needs. One source referred to creating ‘nutrition biographies’ for residents as a way for staff to understand their dietary requirements and eating habits.^31^ However, this responsiveness to resident preferences is also dependent on and can be limited by the foodservice system in place. Particularly in several Swedish studies, a centralized kitchen was supplying food for off‐site elderly care homes, as well as other institutions such as schools.^47^, ^49^ In this context, flexibility to resident needs and changing appetites was reported to be a major challenge. Staff working in centralized kitchens have limited knowledge of the individual residents they are supplying food for and instead rely on communication and ordering from the aged care home, with limited ability to make last minute adjustments to food orders. Similarly, forecasting and preparation of extra serves of food to allow for choice or avoid shortages also makes a significant contribution to production waste before meals are even served. These findings indicate that aged care homes would benefit from adopting more adaptable portioning systems and refined forecasting tools to be able to respond to actual consumption patterns.

While the reduction of food waste can go alongside increased resident satisfaction, as well as economic and environmental benefits, it is important to consider that some level of waste must be acceptable to ensure residents are being offered enough food. Studies in the hospital setting have sought to determine an acceptable level of food waste from hospital foodservices, with one suggesting that 5%–20% plate waste is acceptable depending on the food delivery system.^70^ One source included in this scoping review made a similar statement based on residential aged care, suggesting that 0% plate waste is undesirable as it could mean residents do not get sufficient food, and that plate waste should be between 5% and 20%.^35^ All three studies that applied interventions included in this review resulted in a reduction of plate waste down to between 9% and 15%,^30^, ^31^, ^32^ which is within these ranges and suggests they are reasonable and attainable. As some level of food waste is inevitable, it is important to consider other end‐of‐life strategies for food waste that are in line with the Food Recovery Hierarchy, such as composting or aiming to recover energy from food waste before turning to landfill for disposal.^69^

The heterogeneity in methods of measuring and reporting food waste presents a challenge. While validated visual estimation and weighed measurements were commonly used, missing details on the methods and the lack of standardisation limited comparisons between sources and the synthesis of results. This review found that some studies reported food waste in percentages, while others used varied units of weight or monetary values. Such variability in reporting meant that direct comparisons could not be drawn between sources. However, the approach employed in this review to collate and analyse the data followed best practice methods and still allowed some conclusions to be drawn in an under‐researched field. In the future, standardised approaches to food waste measurement would help to facilitate meta‐analyses or broader comparisons across the literature. Other limitations of this review include the manual data extraction method used, which could result in minor inconsistencies associated with human interpretation. The findings were limited to sources published in English, which excluded sources published in any other language. In the future, research addressing underexplored areas such as production and spoilage waste, and foodservice systems that minimise food waste would contribute to a more holistic understanding of food waste in residential aged care.

This scoping review explored the existing knowledge on food waste in residential aged care and highlights the significant opportunities related to food waste management in this setting. The reduction of food waste has implications that include cost reduction and alignment with sustainability goals, as well as meeting the needs and preferences of residents. The findings of this review suggest that there are a number of potential strategies that could be used to reduce food waste in residential aged care, but that they have not yet been broadly implemented and evaluated. Further research will help to develop a better understanding of food waste in this setting and guide aged care providers to take action to address food waste, which will have positive impacts for aged care residents, the environment, and reduce costs. There is also a clear opportunity for dietitians to lead and contribute to these efforts, drawing on their expertise in nutrition, foodservice systems, and resident‐centred care. Through the prioritization of strategies that consider both resource efficiency and resident satisfaction, aged care homes can contribute to broader sustainability efforts while also improving the quality of their service.

## AUTHOR CONTRIBUTIONS

MR, CT, FP, and DC conceptualized the scoping review, developed the research questions, and developed the search strategy. MR undertook the database and literature searches. MR and DC completed the title and abstract screening and screened the full text for inclusion. MR completed data extraction and drafted the manuscript. All authors reviewed and approved the final draft manuscript for submission.

## FUNDING INFORMATION

This research received no funding.

## CONFLICT OF INTEREST STATEMENT

The authors declare that there is no conflict of interest.

## Data Availability

The data that support the findings of this study are available from the corresponding author upon reasonable request.
